# Supplementary material for: Design of a Composite Based on Polyamide Fabric-Hydrogel-Zinc Oxide Particles to Act as Adsorbent and Photocatalyst
Source: Materials (Basel). 2022 Sep 25;15(19):6649. doi: 10.3390/ma15196649 (PMC9571031; doi:10.3390/ma15196649)
Supplement: Supplementary file 1 [file materials-15-06649-s001.zip › materials-1884765-supplementary.pdf]

Supporting information

Design of a Composite Based on Polyamide Fabric-Hydrogel-Zinc Oxide Particles to Act as Adsorbent and Photocatalyst

Daniela Atanasova [1], Miglena Irikova [1], Desislava Staneva [1,*] and Ivo Grabchev [2,*]

[1] Department of Textile, Leader, and Fuels, University of Chemical Technology and Metallurgy, 1756 Sofia, Bulgaria
[2] Faculty of Medicine, Sofia University St. "Kliment Ohridski", 1407 Sofia, Bulgaria
* Correspondence: grabcheva@mail.bg (D.S.); i.grabchev@chem.uni-sofia.bg (I.G.)

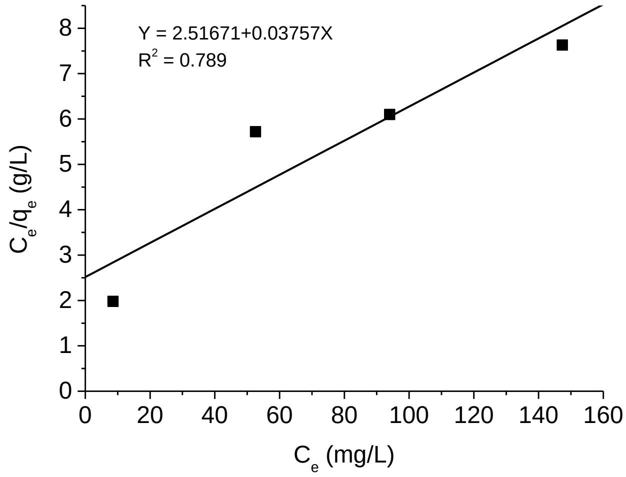

Figure S1. Langmuir adsorption isotherm.

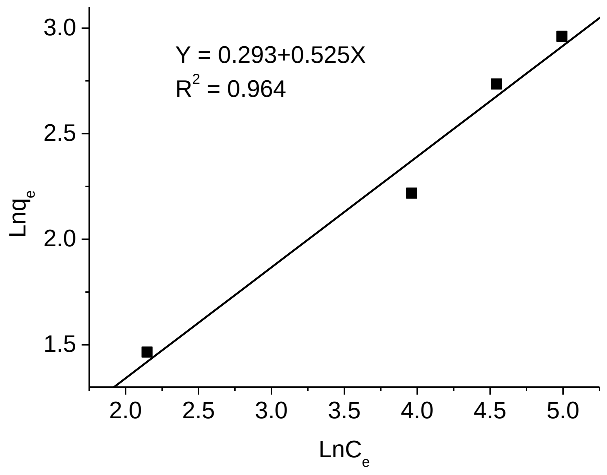

Figure S2. Freundlich isotherm.

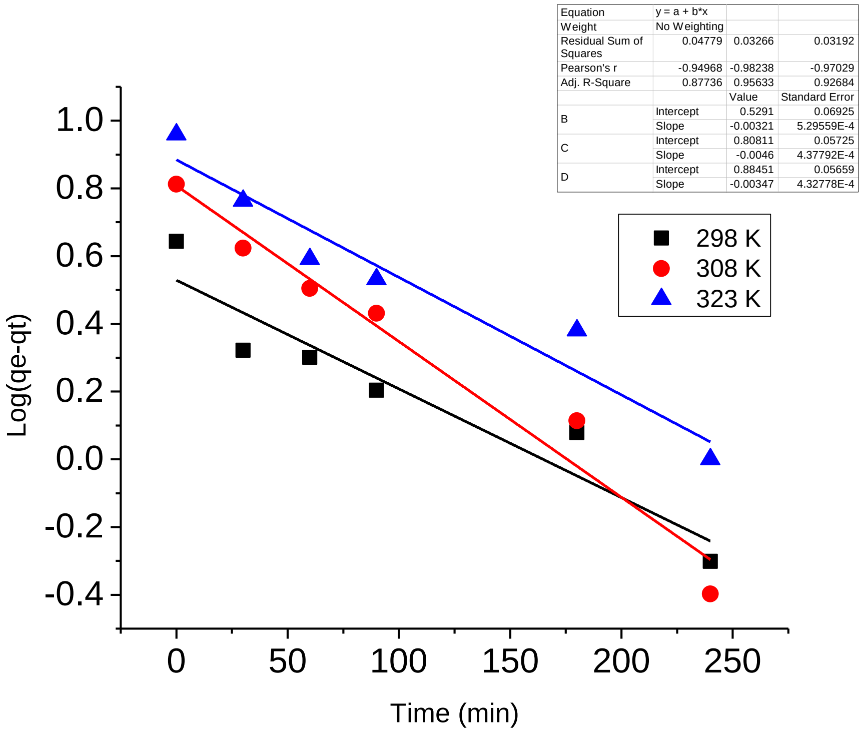

Figure S3. Pseudo-first-order at 298 K, 308 K and 323 K.

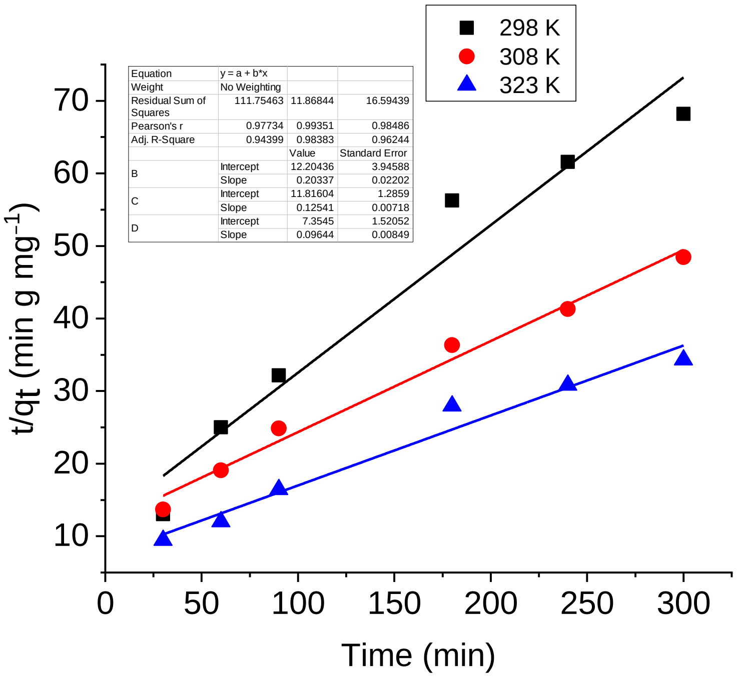

Figure S4. Pseudo-second-order at 298 K, 308 K and 323 K.

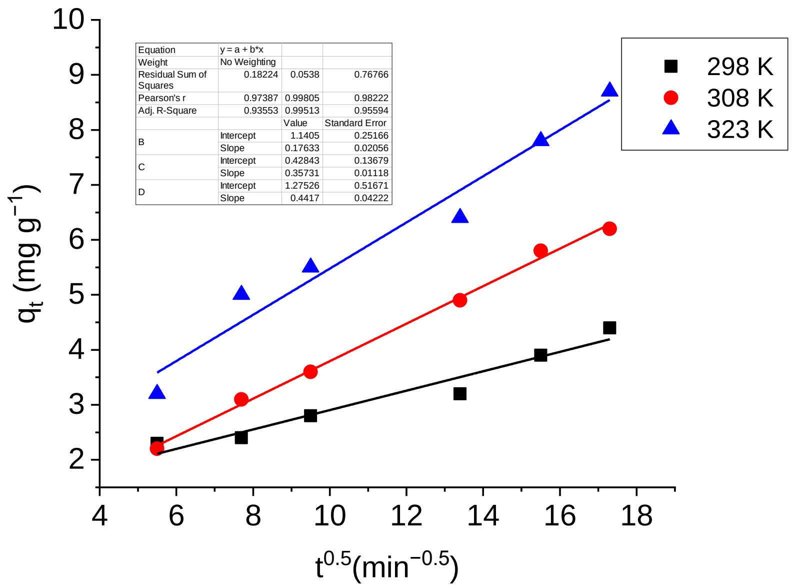

Figure S5. Diffusion kinetic model of the dye adsorption onto composite material PA30 at different temperature.

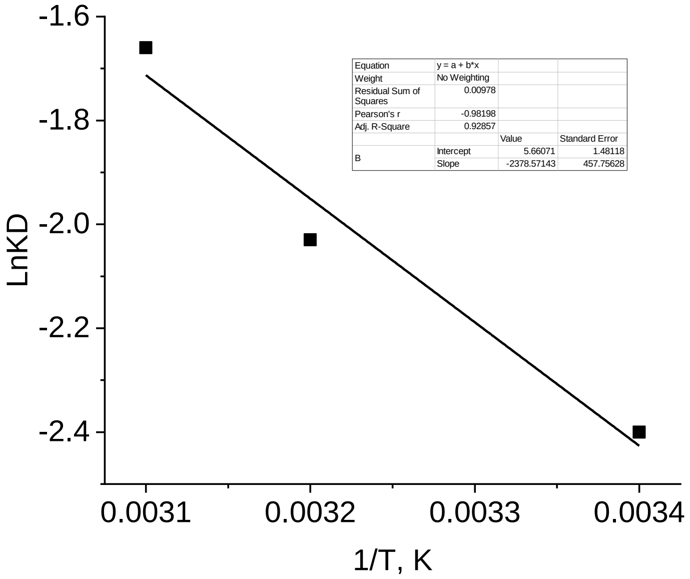

Figure S6. Temperature dependence of the distribution coefficient.

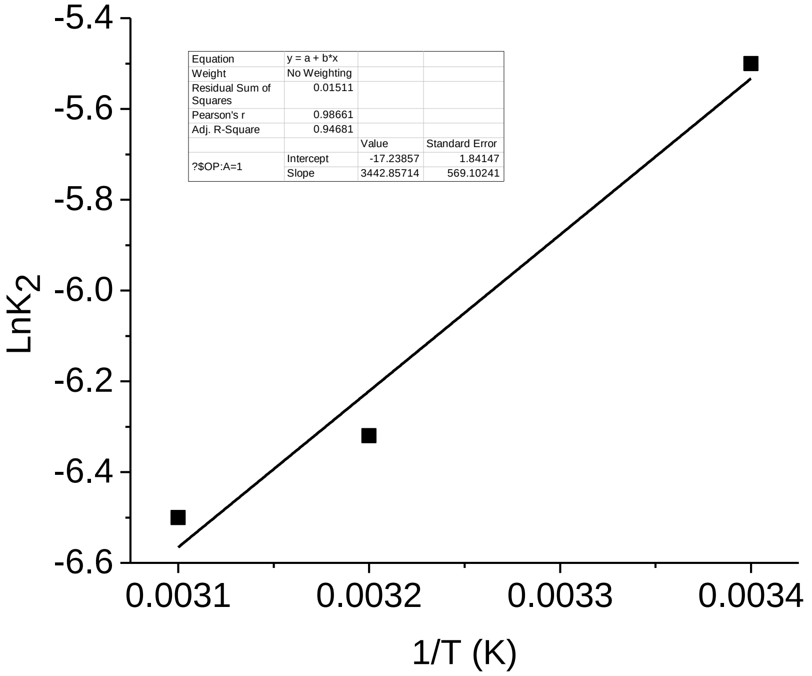

Figure S7. Dependence of LnK_2 on 1/T.

Table S1. Bacterial growth Log (CFU mL $^{-1}$) after incubation with PA30.

Strain	Time of incubation (h)	Log (CFU mL^{-1})		
		**Control**	**Cotton fabric**	**PA30**
S. aureus	1	7.9 ± 0.14	8 ± 0.14	6.33 ± 0.08
	2	7.8 ± 0.05	8 ± 0.08	Full kill
	3	7.7 ± 0.1	7.9 ± 0.13	Full kill
E. coli	1	8.9 ± 0.05	8.8 ± 0.11	7.1 ± 0.05
	2	8.9 ± 0.14	8.9 ± 0.13	7 ± 0.07
	3	8.8 ± 0.1	8.8 ± 0.04	7 ± 0.06
